# Measurement Heterogeneity and Pooled Prevalence of Academic Procrastination Among Health Sciences Students: A Systematic Review and Meta-Analysis

**DOI:** 10.3390/ejihpe16100154

**Published:** 2026-10-01

**Authors:** Víctor Juan Vera-Ponce, Fiorella E. Zuzunaga-Montoya, María Julia Cómina-Tamayo, Darwin A. León-Figueroa, César Iván Guerrero Uceda, Oriana Rivera-Lozada, Jhosmer Ballena-Caicedo, Holly Estrella Delgado-Toro, Mario J. Valladares-Garrido

**Affiliations:** 1 Facultad de Medicina (FAMED), Universidad Nacional Toribio Rodríguez de Mendoza de Amazonas (UNTRM), Chachapoyas 01001, Peru; victor.vera@untrm.edu.pe (V.J.V.-P.); 7330178022@untrm.edu.pe (J.B.-C.); 7694394622@untrm.edu.pe (H.E.D.-T.); 2 Facultad de Medicina, Universidad Continental, Lima 15306, Peru; zuzunaga@continental.edu.pe; 3 Escuela de Medicina Humana, Universidad Peruana Cayetano Heredia, Lima 15102, Peru; maria.comina@upch.pe; 4 EpiHealth Research Center for Epidemiology and Public Health, Lima 15072, Peru; 5 Unidad de Posgrado y Especialización, Tecnologías Avanzadas para la Salud con Enfoque en Inteligencia Artificial, Bioinformática y TIC, Universidad Peruana Cayetano Heredia, Lima 15102, Peru; darwin.leon@upch.pe; 6 Escuela de Medicina Humana, Universidad Señor de Sipán, Chiclayo 14000, Peru; gucedacesarivan@uss.edu.pe (C.I.G.U.); riveraoriana@uss.edu.pe (O.R.-L.); 7 Oficina de Inteligencia Sanitaria, Red Prestacional Lambayeque, EsSalud, Chiclayo 14001, Peru

**Keywords:** procrastination, students, health occupations, prevalence, psychometrics, academic performance

## Abstract

Background/Objectives: In health sciences students, academic procrastination can affect performance, mental health, and professional development. This systematic review and meta-analysis aimed to estimate the pooled prevalence of study-defined elevated academic procrastination among health sciences students and to describe the methodological and measurement characteristics of the instruments used to assess it. Methods: A systematic review with meta-analysis was conducted. We included observational studies reporting the prevalence of academic procrastination among undergraduate students enrolled in health sciences programs, including medicine, nursing, dentistry, psychology, and related health professions. The reported measurement characteristics of the instruments used to assess academic procrastination, including validity, reliability, and cut-off criteria, were reviewed and systematized. The meta-analysis was performed employing a random-effects model and the double arcsine transformation (Freeman–Tukey) to stabilize variance. Subgroup analyses and meta-regressions were calculated to explore possible sources of variation. Results: Twenty-six studies were included with a total of 8211 students. The pooled prevalence of study-defined elevated academic procrastination was 55.35% (95% CI: 46.55–63.98%, I^2^ = 98%). The prevalence was higher in nursing students (62.60%), followed by psychology (61.67%), medicine (52.62%), and dentistry (45.76%). The most commonly used instruments were the Procrastination Assessment Scale for Students (38.5%), Academic Procrastination Scale (23.1%), Tuckman’s Procrastination Scale or its adaptations (15.4%), Academic Procrastination Scale by McCloskey and Scielzo or related APS versions (11.5%), Lay’s Procrastination Scale (7.7%), and MHCA (3.8%). Most studies used non-probabilistic sampling, and only 8% validated instruments specifically for their target populations. Reliability coefficients were generally adequate (α = 0.75–0.95). The meta-regression revealed a slight upward trend in prevalence as a function of publication year, although this was not statistically significant. Conclusions: Academic procrastination appears to be frequent among health sciences students; however, the pooled estimate should be interpreted as a descriptive summary of study-defined prevalence rather than as a universal prevalence estimate, given the very high between-study heterogeneity and the variability in instruments, cut-off criteria, and population-specific validation. Educational institutions should implement prevention and support strategies, while research should prioritize instrument validation, measurement standardization, and transparent case definition criteria.

## 1. Introduction

Academic procrastination has emerged in recent years as a significant behavioral phenomenon in the university environment, recognized as a form of failed self-regulation with important consequences for academic performance, mental health, and quality of life of students (Araya-Castillo et al., 2023; Rozental et al., 2022). Procrastination is commonly defined as the voluntary delay of an intended course of action despite expecting to be worse off because of the delay (Steel, 2007). In the academic context, this definition implies not merely postponement, but the delay of an intended academic task despite anticipated academic, emotional, or functional consequences. At the university level, this behavior can affect between 50% and 70% of students, thus representing one of the main barriers to proper academic development; furthermore, its association with anxiety and depressive symptomatology has been documented by various studies (Svartdal et al., 2020). Regarding its magnitude and negative effects, procrastination represents a behavioral problem of public health importance that warrants systematic study to understand its true scope better (Rusdi et al., 2020).

The negative consequences of academic procrastination extend beyond immediate academic performance, creating a cascade of detrimental effects that can persist throughout the academic trajectory and into professional life. Research demonstrates that procrastinating students experience significantly higher levels of stress, anxiety, and depressive symptoms compared to their non-procrastinating peers (Pychyl & Flett, 2012). Academic procrastination is also associated with poorer sleep quality, increased substance use, and compromised physical health, including higher rates of illness and weakened immune function (Sirois & Pychyl, 2013). From an academic perspective, procrastination leads to lower grade point averages, increased course withdrawals, and higher dropout rates, with some studies reporting that chronic procrastinators are 1.5 times more likely to leave university prematurely (Steel & Klingsieck, 2016). Furthermore, the economic impact is substantial, as procrastination-related academic delays result in increased tuition costs and delayed entry into the workforce, creating long-term financial burdens for both students and their families (Howell & Watson, 2007). In health sciences education specifically, procrastination can compromise the development of critical professional competencies, potentially affecting future patient care quality and professional confidence (Seo, 2009).

On the other hand, among the determinants most frequently observed in relation to procrastination are maladaptive perfectionism, low intrinsic motivation, deficits in self-regulation skills, and excessive use of social networks and digital technologies (X. Zhou et al., 2024). This phenomenon not only affects academic performance but also involves psychological well-being and prolongation of university graduation time, generating a negative economic impact for both students and higher education institutions (Akdeniz & Duygulu, 2025; Desai et al., 2021).

In recent years, there has been an increase in evidence related to the actual prevalence of procrastination in university students; however, there is heterogeneity in the measurement methods used, which makes comparisons between studies difficult (Albursan et al., 2022; Shukla & Andrade, 2023; Y. Zhou et al., 2024).

Differences in cultural contexts, educational levels, professional programs, and measurement approaches have generated heterogeneous and sometimes inconsistent prevalence estimates, making it difficult to establish reliable and comparable estimates of academic procrastination (Limone et al., 2020). Although academic procrastination has been widely studied in university populations, evidence focused specifically on health sciences students remains fragmented. This group faces distinctive academic pressures, clinical training demands, and professional competency expectations. From a public health perspective, academic procrastination among health sciences students is relevant because it may affect psychological well-being, academic progression, professional competency development, and the future health workforce. Moreover, prevalence estimates vary widely due to differences in instruments, cut-off points, and population-specific validation. Understanding both its magnitude and measurement limitations is therefore important for designing preventive, educational, and mental health support strategies in higher education settings. Therefore, this systematic review and meta-analysis aimed to estimate the pooled prevalence of academic procrastination among health sciences students and to examine how measurement characteristics contribute to heterogeneity across studies.

## 2. Materials and Methods

### 2.1. Study Design

A systematic review with meta-analysis of observational studies reporting the prevalence of academic procrastination among health sciences students was conducted. The methodological guidelines of the PRISMA 2020 statement (Preferred Reporting Items for Systematic Reviews and Meta-Analyses) were followed (Page et al., 2021). The PRISMA checklist is provided in Table S1. This review was not prospectively registered, and no separate review protocol was prepared; no clinical trial registration number is applicable.

### 2.2. Search Strategy

The bibliographic search strategy was carried out in four electronic databases with broad recognition and international coverage—PubMed/MEDLINE, Scopus, Web of Science (including Scielo), and EMBASE—covering the period from 2000 to 2025. The selection of these databases took into account the Cochrane guidelines, ensuring thematic and geographical coverage. The search combined controlled descriptors (MeSH) and free terms relevant to the study topic such as “academic procrastination”, “procrastination”, “college students”, “university students”, “prevalence”, “higher education”, and “delay behavior”. Boolean operators (AND, OR) were used to structure the search and combine related terms. The search was complemented by a manual review of reference lists from key articles. The complete strategy is presented in Table S2.

### 2.3. Selection Criteria

Studies included in this review were those that had an observational design, including cross-sectional, cohort, or prospective studies, and that reported point or period prevalence of academic procrastination among undergraduate students enrolled in health sciences programs. Eligible programs included medicine, nursing, dentistry, psychology, and related health professions. Studies including general university students were eligible only when data for health sciences students were reported separately or could be extracted. Only studies that used validated psychometric instruments or clear operational definitions to measure academic procrastination were included. Additionally, studies published in English, Spanish, and Portuguese were accepted, provided that the full text was available and allowed extraction of the necessary data.

In contrast, studies that did not directly report prevalence data or that focused on non-university populations, such as school students, working adults, or clinical populations, were excluded. Studies involving general university populations without extractable data for health sciences students were also excluded. Case reports, qualitative studies, systematic reviews, narrative reviews, letters to the editor, editorials, and bibliometric studies were excluded.

### 2.4. Selection Process

All search results were exported in RIS format and uploaded to the Rayyan platform (https://new.rayyan.ai/; accessed on 15 April 2026). The first step consisted of automatic elimination of duplicates, followed by a manual review to verify matches not detected by the system. Subsequently, two independent reviewers blindly evaluated the titles and abstracts of the identified studies to determine their eligibility. Articles that met the criteria were selected for full-text review. This second phase was also conducted by two researchers independently; if there were discrepancies between reviewers, they were discussed case by case, and if an agreement could not be reached, the decision of a third reviewer with methodological expertise was sought.

### 2.5. Data Collection Process

Data extraction was performed by two researchers independently using a structured template designed in Microsoft Excel 2023, which was previously piloted on a sample of five studies to ensure its functionality. Extracted data included the name of the first author, year of publication, country where the study was conducted, methodological design, type of university population, sample size, proportion of women, mean age, sampling method, instrument used to measure procrastination, and operational definitions employed, as well as point estimates of total prevalence and, if available, whether the studies were stratified by sex or other variables. Secondary outcomes such as prevalence by severity levels, associated factors, and presence of psychological comorbidities were also collected. Both reviewers verified all information, and inconsistencies were resolved by re-examining the original text or by consensus.

### 2.6. Study Risk of Bias Assessment

The risk of bias of the included studies was evaluated using the prevalence study tool modified by Hoy et al. (2012), specifically adapted for prevalence studies in public health. This tool assesses 10 methodological domains, including sample representativeness, accuracy of the sampling frame, adequacy of recruitment methods, quality of measurements, clear definition of the studied condition, instrument validity, and statistical analysis quality. Each domain was classified as “low,” “high,” or “uncertain” risk. Two reviewers applied the tool independently, and disagreements were resolved through discussion. A total score was assigned to each study, categorizing them as low risk (8 to 10 points), moderate risk (5 to 7 points), or high risk (less than or equal to 4 points). This evaluation was used both to interpret the results and to perform sensitivity analysis in the meta-analysis.

### 2.7. Statistical Analysis

Statistical analysis was performed in the R environment (version 4.3.1), mainly using the meta, metafor, and ggplot2 packages. For the meta-analysis, only studies that explicitly reported the total number of participants and an extractable number of academic procrastination cases were included. When studies reported a dichotomous prevalence, this numerator was used directly. When studies reported ordered severity categories, the numerator was extracted according to a predefined harmonization rule: participants classified as moderate, high, severe, or equivalent levels were coded as cases, whereas participants classified as low, mild, absent, or non-procrastination were coded as non-cases. This approach preserved the primary authors’ instrument-specific thresholds and avoided imposing a single post hoc threshold across scales that were not metrically equivalent. For studies using latent profiles or categorical severity solutions, categories explicitly labeled by the original authors as moderate or high procrastination were treated as study-defined cases. This operational decision should not be interpreted as identifying chronic procrastinators; rather, it identifies participants meeting each study’s threshold for educationally relevant or elevated academic procrastination. When a primary study reported both high-only and moderate-to-high categories, the moderate-to-high numerator was used for the main synthesis if consistent with the predefined rule, and the original high-only threshold was documented in the instrument characteristics table. Studies without extractable numerators or without a sufficiently clear operational definition were excluded from the quantitative synthesis.

The Freeman–Tukey double arcsine transformation stabilized the variance of proportions, and 95% confidence intervals were estimated using the Wilson Score (WS) method. A random-effects model was applied, with estimation of between-study variance (τ^2^) according to the DerSimonian–Laird method and standard error correction with the Hartung–Knapp approximation to improve precision in contexts of high heterogeneity.

Heterogeneity between studies was evaluated using Cochran’s Q test and the I^2^ index, with interpretation according to conventional criteria. Forest plots were developed, grouped by variables such as country, measurement instrument, professional career, sample design, and publication decade. Additionally, subgroup analyses and univariate and multivariate meta-regression were carried out using the rma() function of the metafor package, exploring the influence of continuous variables (such as year of publication) and categorical variables (instrument used, type of sampling, professional career, geographic region) on the prevalence estimate. The results of the meta-regression were represented in bubble plots, in which the size of each point reflects the sample size of the study.

Additionally, a global prevalence map was generated using the rworldmap and ggplot2 packages, with visualization by country. Sensitivity analyses were also performed, excluding studies with methodological risk or incomplete information, to evaluate the robustness of global estimates.

## 3. Results

### 3.1. Process of Selected Articles

The PRISMA flow diagram illustrates the systematic review process for studies on the prevalence of academic procrastination. Initially, 4199 records were identified through database searches (Scopus: 1879, Embase: 356, PubMed: 362, Web of Science: 1602), with no additional records from other sources. After removing duplicates, 2436 records remained for screening, of which 2390 were excluded mainly for not evaluating the prevalence of procrastination, including non-university populations, or using non-observational designs. Figure 1 shows the PRISMA flow diagram. Forty-six full-text articles were assessed for eligibility, with 20 being excluded due to inadequate prevalence data, unvalidated instruments, or inaccessibility. Finally, 26 studies were included in both the qualitative synthesis and the meta-analysis (see Table S3; included studies are marked with an asterisk in the reference list).

### 3.2. General Characteristics of Included Studies

The included studies were published between 2012 and 2024, with a notable increase in publications from 2020 onward, a period that accounted for 84.6% of the studies (*n* = 22/26) (Table S3). The included studies were published between 2012 and 2024, with a notable increase in publications from 2020 onward, a period that accounted for 84.6% of the studies (*n* = 22/26) (Table S3). The studies included in the systematic review and meta-analysis were: Madhan et al. (2012), Atalayin et al. (2018), Custer (2018), Khalid et al. (2019), Hayat et al. (2020a), Chávez Parillo et al. (2021), Kafipour and Jafari (2021), Fentaw et al. (2022), Orco León et al. (2022), Tahir et al. (2022), Awad et al. (2023), Estrada-Araoz et al. (2023), García-Álvarez et al. (2023), Moya-Salazar et al. (2023), Real-Delor et al. (2023), Ali et al. (2024), Ghasempour et al. (2024), Ghattas and El-Ashry (2024), and Thapa and Rimal (2024). Regarding methodological design, all included studies were cross-sectional observational studies. Twenty-four studies (92.3%) employed non-probabilistic sampling methods, while two studies (7.7%) used probabilistic sampling. Sample sizes showed considerable variability, ranging from 44 to 1369 participants. The median sample size was 255 participants, with an estimated interquartile range between 140 and 452 students.

The studied populations consisted exclusively of undergraduate students enrolled in health sciences programs, including medicine, nursing, dentistry, psychology, and mixed or unspecified health sciences programs (Table S3). Studies that included students from more than one health sciences program without program-specific data were classified as mixed health sciences. Most studies included participants of both sexes; among studies reporting sex distribution, the proportion of women exceeded 50% in most cases. The reported mean age ranged from 18.9 to 29 years, with most studies reporting mean ages between 20 and 23 years. When the study setting was described, populations were predominantly urban and university-based.

The risk of bias assessment showed that 25 studies (96.2%) obtained the maximum score of 8 points and were classified as having low risk of bias. One study (3.8%) scored 7 points and was classified as having moderate risk of bias due to limited clarity in the description of the study population. It is noteworthy that the two studies that employed probabilistic sampling obtained the maximum score, with sampling design being one of the few domains that contributed variability to the overall rating. Common methodological strengths included the use of validated instruments to measure academic procrastination and clear reporting of inclusion and exclusion criteria. Among the most frequent methodological limitations was the predominant use of non-probabilistic sampling and incomplete reporting of response rates or strategies to control for non-response bias.

These ratings should be interpreted as risk of bias within the domains assessed by the prevalence study tool, not as evidence of population representativeness. Non-probability sampling was penalized within the sampling and representativeness domain; however, several studies still obtained an overall low-risk classification because they used clearly defined populations, standardized measurement instruments, explicit eligibility criteria, and adequate statistical reporting. Consequently, low risk of bias does not eliminate concerns regarding external validity or generalizability.

### 3.3. Meta-Analysis of Study-Defined Academic Procrastination Prevalence

The prevalence of study-defined elevated academic procrastination reported in the included studies ranged from 18.18% to 77.5%. The general meta-analysis, based on 26 studies with a total of 8211 participants, yielded a pooled prevalence of 55.35% (95% CI: 46.55–63.98%) (see Table S3), with significant heterogeneity between studies (I^2^ = 98%, *p* < 0.001). Given the very high heterogeneity, this estimate should be interpreted as a descriptive summary of study-defined prevalence rather than as a definitive universal prevalence estimate.

In analyses by type of sampling, studies with non-probabilistic design (*n* = 24) reported a combined prevalence of 53.44% (95% CI: 44.30–62.47%, I^2^ = 98%), while the two studies with probabilistic sampling reported a higher prevalence of 77.46% (95% CI: 68.32–85.46%), with less heterogeneity (I^2^ = 73%).

Regarding the instrument used to evaluate procrastination, relevant variations were observed. Studies using the PASS questionnaire (*n* = 10) reported a combined prevalence of 60.10% (95% CI: 43.34–75.74%, I^2^ = 98%). Studies using the EPA instrument (*n* = 6) showed a prevalence of 45.38% (95% CI: 29.10–62.17%, I^2^ = 98%), while studies using the APS instrument (*n* = 3) reported a prevalence of 60.84% (95% CI: 28.43–88.66%, I^2^ = 99%). The TPS-TV instrument or its adaptations (*n* = 4) showed a prevalence of 65.69% (95% CI: 42.62–85.42%, I^2^ = 98%). Studies using Lay’s Procrastination Scale (LPS; *n* = 2) showed a pooled prevalence of 46.58% (95% CI: 11.89–83.31%, I^2^ = 99%), whereas the single study using MHCA reported a prevalence of 26.61% (95% CI: 19.62–35.01%).

In the analysis by type of program, studies conducted on nursing students (*n* = 8) presented the highest combined prevalence with 62.60% (95% CI: 46.88–77.08%, I^2^ = 98%). They were followed by psychology students (*n* = 2) with a prevalence of 61.67% (95% CI: 20.95–94.42%, I^2^ = 99%). Studies on medical students (*n* = 12) reported a prevalence of 52.62% (95% CI: 37.63–67.38%, I^2^ = 98%), while studies on dental students (*n* = 4) indicated a combined prevalence of 45.76% (95% CI: 31.64–60.23%, I^2^ = 97%); see Table 1.

### 3.4. Instruments Used for the Assessment of Procrastination

Six different instruments were identified for evaluating academic procrastination in the 26 included studies (Table 2). The most commonly used instrument was the Procrastination Assessment Scale for Students (PASS), used in 10 studies (38.5%), followed by the Academic Procrastination Scale (EPA) in 6 studies (23.1%), the Tuckman Procrastination Scale or its adaptations in 4 studies (15.4%), the Academic Procrastination Scale by McCloskey and Scielzo or related APS versions in 3 studies (11.5%), Lay’s Procrastination Scale (LPS) in 2 studies (7.7%), and the MHCA scale in 1 study (3.8%).

Regarding psychometric validation for the specific study populations, only two studies (8%) conducted formal validation procedures specifically for their target populations: Corrales-Reyes et al. (2022) validated the EPA-11-Odonto scale in Cuban stomatology students, and Portillo and Soto (2024) designed and validated the MHCA scale in Mexican medical students. The remaining 24 studies (92%) used instruments previously validated in other populations or cultural contexts but did not perform specific validation for their study populations; therefore, the reported prevalence estimates should be interpreted in light of possible measurement transferability limitations.

Among the studies using previously validated instruments, most relied on established scales such as Solomon and Rothblum’s (1984) PASS, Lay’s (1986) Procrastination Scale, McCloskey and Scielzo’s validation, or on cultural adaptations such as Domínguez’s Peruvian adaptation. However, several studies did not report clear information about the validation background of their chosen instruments (Ahmed et al., 2023; Manu et al., 2024; Turki et al., 2023).

Reliability assessment for the study samples was reported in 22 studies (85%), with internal consistency measured by Cronbach’s alpha being the most frequently used method. Reported Cronbach’s alpha values ranged from 0.75 to 0.95 across the included studies, whereas four studies did not report reliability coefficients. The study-specific reliability information is summarized in Table 2.

Cut-off points for defining significant or elevated procrastination varied considerably among instruments and studies. Studies used absolute score thresholds, median splits, percentile-based classifications, upper-tertile classifications, or predefined categorical levels such as low, moderate, high, severe, or very high. Several studies did not specify clear cut-off criteria and either treated procrastination as a continuous variable or failed to provide classification thresholds. This variability in cut-off criteria represents an important methodological consideration when comparing prevalence estimates across studies (Table 2).

### 3.5. Analysis of Study-Defined Academic Procrastination Prevalence by Country

Prevalence data grouped by country were analyzed to visualize geographical differences in study-defined academic procrastination among health sciences students; see Figure 2. Prevalence estimates showed high variability between countries, with ranges oscillating between 22.7% in the United Arab Emirates and 87.2% in Tunisia. Other countries with high prevalences were Egypt (85.9%), Ethiopia (81.1%), and Iran (76.5%). In contrast, the lowest values were reported in Mexico (26.6%) and Cuba (39.6%).

In the Americas, prevalences were heterogeneous: the United States presented one of the highest prevalences in the continent (71.3%), while Venezuela (40.0%), Peru (41.1%), and Paraguay (38.8%) showed intermediate values. In Asia, values above 60% were observed in China (67.5%) and Malaysia (66.5%). Meanwhile, India, Pakistan, and Nepal showed more moderate prevalences (53.1%, 49.1%, and 40.1%, respectively). Meanwhile, Pakistan and Nepal showed more moderate prevalences (49.1% and 40.1%, respectively). Most African and Middle Eastern countries included in the sample presented the highest proportions of the analysis; this can be seen in Figure 2.

### 3.6. Meta-Regression Analysis of Procrastination Prevalence According to Year of Publication

A meta-regression with the year of publication was performed to explore possible temporal trends in the prevalence of academic procrastination among health sciences students. Figure 3 shows the results of this analysis. The regression slope was positive, indicating a slight increase in reported prevalences over time. However, this association did not reach statistical significance, suggesting that, although there is an apparent trend of increase in the observed values, this could be attributed to the variability between studies rather than a consistent temporal pattern.

Visually, the bubble plot shows considerable dispersion of prevalence estimates over the years, with a greater concentration of studies between 2020 and 2024. The shaded area represents the confidence interval of the regression model, which is wide and reflects the uncertainty of the estimate. The red dashed line represents the estimated trend, pointing toward a slight increase in the average reported prevalence of procrastination. The larger bubbles correspond to studies with larger sample sizes, and the colors identify geographical origin, evidencing a wide diversity of cultural and educational contexts over time; see Figure 3.

## 4. Discussion

### 4.1. Interpretation and Possible Explanations

Academic procrastination is extremely common among university students, affecting most students in various contexts (Gómez-Romero et al., 2020). In health sciences programs, this high prevalence is related to the intense academic load and stress that students face. Recent studies indicate that medical and nursing students report moderate to high levels of procrastination, frequently postponing tasks until the last moment (Cao et al., 2025). High academic stress and pressure for performance contribute to this phenomenon; in fact, a strong correlation has been observed between the perception of stress and the tendency to procrastinate in this group of students (Cao et al., 2025). Additionally, technological distractions play an important role: the widespread use of the internet and social networks has been linked to greater procrastination in medical students, reflected in the fact that approximately 29% present high academic procrastination significantly associated with internet addiction (Hayat et al., 2020b).

Beyond external demands, there are inherent psychological factors that explain why so many health sciences students procrastinate. Procrastination is deeply rooted in difficulties of emotional self-regulation and cognitive biases (Portillo & Soto, 2024). Many students in health areas are perfectionists and fear failure, which paradoxically leads them to postpone tasks to avoid the anxiety of not meeting their high expectations. This emotional pattern creates a negative cycle: the initial delay generates guilt and anxiety, which further reinforces procrastination and can lead to symptoms of depression. In fact, a positive association between academic procrastination and depression has been documented in health sciences students (Nazari et al., 2021). Likewise, many report feeling exhausted and demotivated, especially at the beginning of their medical career, favoring the postponement of academic activities (Portillo & Soto, 2024). In summary, the combination of high academic loads, chronic stress, and emotional factors (perfectionism, anxiety, and fatigue) explains why academic procrastination is so prevalent in health sciences students.

### 4.2. Factors Explaining Differences by Region, Instrument, and Program

Various instruments have been developed to measure procrastination in the academic field, each with different structures and psychometric evidence. The PASS by Solomon and Rothblum (1984) is one of the first and most used; it consists of two parts: frequency of postponement in six academic tasks and possible reasons for delay. Although the PASS lacked an original alpha coefficient, subsequent studies report internal consistencies from 0.66 to 0.85 (Martín-Antón et al., 2023). Its initial factor analysis suggested two dimensions of motives, those being fear of failure and task aversion, although later adaptations have shown different structures across contexts (Solomon & Rothblum, 1984; Vangsness et al., 2022). In contrast, Busko’s (1998) EPA—also known as the Academic Procrastination Scale—is a brief Likert-type instrument originally described as unidimensional. This scale, widely applied in Spanish-speaking countries, shows satisfactory properties but has revealed variable factorial structures depending on the sample: some studies support two factors, such as procrastination in daily study versus exam preparation, whereas others find broader related factors such as task aversion, poor time management, low emotional-motivational self-control, and risk-seeking (Busko, 1998; Fior et al., 2022). Meanwhile, the TPS is conceived as a unidimensional measure of behavioral delay in academic tasks and has shown favorable psychometric performance in several adaptations (Galindo-Contreras & Olivas-Ugarte, 2022; Tisocco & Fernández Liporace, 2021; Tuckman, 1991).

An important conceptual issue is that the theoretical definition of procrastination contains at least two core elements—voluntary delay and expected harm—whereas many instruments primarily measure frequency of delay, task aversion, poor planning, or self-regulatory difficulty. Therefore, a high score on a procrastination scale is not always equivalent to meeting the full theoretical definition of procrastination. This discrepancy is directly relevant to prevalence research: studies may estimate the proportion of students above a scale threshold, not necessarily the proportion of chronic procrastinators. Accordingly, the pooled prevalence in this review should be interpreted as the prevalence of study-defined elevated academic procrastination rather than as a uniform diagnostic- or trait-based prevalence.

Subsequently, McCloskey and Scielzo (2015) developed the 25-item APS, based on six theoretical facets of the procrastinator: beliefs about ability, distraction, social factors, time management, personal initiative, and laziness. Although it covers multiple content dimensions, empirically, the APS was essentially unidimensional, with excellent internal consistency. Additionally, it showed strong concurrent validity, correlating highly with PASS, TPS, and related instruments, and predictive validity, significantly associating with worse academic performance (McCloskey & Scielzo, 2015; Vangsness et al., 2022). Regarding the measurement of academic procrastination, it is carried out using various instruments such as LPS, which evaluates the tendency to postpone in general; the MHCA scale developed in Mexico, which categorizes procrastinating behaviors in six areas through 17 dichotomous items; and other scales that vary in length, response format, and conceptual approach. Although these instruments show acceptable levels of reliability, they differ significantly in their structure, generating only moderate correlations between them as they capture different nuances of the same phenomenon (Argumedo et al., 2005).

The lack of population-specific validation is one of the most important methodological limitations identified in this review. Although most studies reported acceptable internal consistency, reliability alone does not ensure construct validity, measurement invariance, or appropriate cut-off performance in a specific academic, cultural, or professional context. Therefore, prevalence estimates based on instruments validated in other populations may reflect measurement transferability problems rather than true differences in procrastination burden.

Each instrument’s different approaches and properties carry important limitations and controversies that affect the comparison of results between studies, especially in prevalence estimates. The first point lies in the conceptual structure. For example, the PASS combines frequency and reasons for delay, complicating its scoring and leading to inconsistent factorial results according to culture and educational level (Martín-Antón et al., 2023; Solomon & Rothblum, 1984). Similarly, Busko’s EPA has oscillated between unifactorial and multifactorial solutions depending on the sample, suggesting that the manifestation of procrastination may vary between contexts; for example, some studies separate procrastination in daily studies from procrastination in exam preparation (Caceres-Ravelo et al., 2024; Fior et al., 2022; Martín-Antón et al., 2023). This variability indicates possible cultural or language influences in the interpretation of items, making the instruments vulnerable to cultural differences (Svartdal et al., 2016). In contrast, very specific or unidimensional scales such as the TPS may oversimplify the phenomenon by focusing mainly on behavioral delay and not fully capturing motivational, affective, or functional dimensions; however, their clarity may favor consistency at the cost of not capturing the underlying reasons for delay. Another controversy is the scope of the measured construct: Lay’s LPS evaluates procrastination as a general trait, so a student could obtain a high score for postponing non-academic tasks, inflating their level without necessarily reflecting academic procrastination (Lay, 1986). Conversely, an instrument focused only on academic tasks could overlook the individual’s tendency to delay obligations. Furthermore, there are differences in the criteria for defining someone as a “procrastinator” according to the instrument and the author. While some works treat procrastination as a continuous variable, others define thresholds to categorize its prevalence. For example, using the Lay scale, a significant procrastinator has been considered as one who scores ≥ 60 (out of 100); in contrast, Uma et al. used the same Lay scale but defined high procrastination as ≥62, while the present synthesis used moderate plus high categories according to the predefined harmonization rule. Across instruments, this problem is amplified because severity categories are not directly comparable, and many students acknowledge delaying academic tasks although not all do so chronically.

On the other hand, dichotomous instruments such as the MHCA tend to generate higher prevalences of “procrastinators” because each affirmative item adds to the count (≥5 “yes” items). In contrast, Likert scales consistently require high scores across the entire scale to qualify someone at the upper extreme of procrastination, producing more conservative estimates. These methodological discrepancies (number of items, cut-off criteria, evaluated content) largely explain the heterogeneity between studies of academic procrastination prevalence. In other words, the proportion of students identified as procrastinators varies widely depending on whether the instrument emphasizes delay frequency, problem perception, specific context, or the threshold chosen to define “significant procrastination.” Therefore, caution has been suggested when comparing results and selecting the measurement tool (Vangsness et al., 2022). In this way, researchers should ensure that the instrument used has solid psychometric properties in their sample and be transparent about how they define academic procrastination in operational terms. Only then can differences in prevalence be attributed more to true population variations than measurement artifacts. Ultimately, understanding the strengths and limitations of each instrument—from its reliability and validity to its theoretical approach—helps to interpret the literature more critically, explaining why the prevalence of academic procrastination can vary widely and highlighting the need for greater standardization in assessing this phenomenon.

Severity classifications should ideally consider not only the frequency or intensity of academic delay but also functional impairment and psychological correlates such as anxiety, depressive symptoms, stress, sleep problems, or academic dysfunction. However, these variables were not consistently reported across the included studies; therefore, they could not be incorporated into the case definition without introducing additional reporting bias. Future prevalence studies should distinguish occasional academic delay from clinically or educationally relevant procrastination by combining scale scores with impairment, distress, and functional consequences.

Taken together, these methodological discrepancies indicate that heterogeneity should not be interpreted only as statistical variability. A substantial part of the observed heterogeneity is likely measurement-driven, arising from non-equivalent instruments, inconsistent thresholds, limited population-specific validation, and differences in whether studies captured occasional delay, elevated academic procrastination, or more persistent procrastinator status. This distinction is essential when translating prevalence estimates into educational or mental health policies.

### 4.3. Implications for Practice and Research

The pooled estimate should be interpreted primarily as a descriptive synthesis of study-defined prevalence, not as a definitive universal estimate. The very high heterogeneity indicates that most variability likely arises from between-study differences rather than sampling error alone. Therefore, the overall prevalence is less informative than the pattern of heterogeneity itself, which points to differences in instruments, cut-off points, sampling procedures, academic programs, and cultural contexts. From a practical perspective, the finding supports the relevance of academic procrastination in health sciences education, but it should not be used as a single benchmark for institutional prevalence without local validation. This has important implications for universities, health sciences faculties, and training programs, which should consider academic procrastination as a potential barrier to performance, mental health, and academic progression. For teachers, these results imply the need to go beyond content delivery, adopting a pedagogical approach that integrates emotional self-regulation skills and time management. Continuous assessments, tutorial support, and active learning environments can be key tools to mitigate this behavior in the classroom.

From an institutional perspective, universities must recognize that procrastination is not only an individual problem but also a structural one. The highly competitive academic culture, task overload, and limited curricular flexibility can reinforce postponement patterns. Thus, it is recommended that institutions implement comprehensive academic and psychological support programs, including personalized counseling, study skills training, stress management, and coping techniques. Early identification of students with high procrastination can also be part of academic alert systems, helping to prevent educational failure or dropout.

In terms of educational psychology, the results demonstrate the need to incorporate specific interventions for procrastination as part of university mental health services. Therapies based on self-regulation, such as Cognitive–Behavioral Therapy, training in various areas, or motivation-based interventions, have proven effective in reducing procrastination in academic contexts. Additionally, it has been observed that brief programs aimed at improving time management and planning can have positive effects, especially when personalized according to the student’s learning style. Psychologists should consider procrastination not only as an isolated behavior but as a symptom that may be associated with other problems such as anxiety, depression, or low self-esteem.

In terms of future research, the findings of this review suggest the need to design longitudinal studies that allow an understanding of how procrastination evolves throughout the university career and what personal, academic, or institutional factors aggravate or mitigate it. Furthermore, it is important to promote research with mixed methodologies, combining quantitative prevalence analyses with qualitative studies that explore in depth the reasons and experiences behind procrastinatory behavior. Studies that validate and compare assessment instruments across different cultures are also required to advance toward a more standardized and sensitive measurement of the phenomenon.

Finally, at the level of educational policies, these results could inform the design of preventive strategies from the first university year, especially in demanding programs such as medicine or nursing. Including content on self-care, emotional regulation, and study habits in induction courses or transversal subjects can sustain and reduce procrastination. Likewise, the findings can be useful for reviewing institutional assessment practices, promoting more balanced academic calendars, teaching practices that reinforce intrinsic motivation, and student monitoring models centered on well-being. Together, this evidence offers a solid basis for educational actors to work in a coordinated manner in preventing and managing academic procrastination in health sciences.

### 4.4. Strengths and Limitations

This SR and meta-analysis present several notable methodological strengths. First, an exhaustive search was conducted in multiple databases, and rigorous inclusion criteria were applied to ensure the quality and relevance of the selected studies. Most included studies presented a low risk of bias, reinforcing the estimates’ reliability. Likewise, a robust statistical approach was used, applying a random-effects model, the Freeman–Tukey transformation to stabilize variance, and the Hartung–Knapp correction, which allowed for the adequate management of the observed heterogeneity. Subgroup analyses and meta-regressions were also explored to identify possible sources of variability, and sensitivity and publication bias analyses were incorporated. Finally, this review offers a comprehensive and updated synthesis of the phenomenon of academic procrastination, specifically in health sciences students, a group poorly addressed in previous reviews, which provides novel and specific evidence for this field.

However, this review also presents some limitations that should be considered when interpreting the results. First, most studies employed non-probabilistic sampling, which limits the representativeness of the studied populations. The heterogeneity observed between studies was very high, and although sources of variability were explored through subgroups and meta-regressions, unmeasured factors—such as cultural differences, academic contexts, collection calendar, teaching style, and local assessment practices—may also have influenced the results. Additionally, our search was limited to English, Spanish, and Portuguese languages, which may have introduced language bias, though no relevant studies in other languages were identified during our comprehensive database search. Another critical point is the diversity of instruments used and the fact that most studies (92%) used instruments that were not specifically validated for their target populations, along with inconsistent cut-off points across studies, complicating the comparison between prevalence estimates. Finally, not all studies reported complete information on diagnostic criteria or the distribution of procrastination levels, which may have restricted the analysis of some subgroups. Although this review followed PRISMA 2020 recommendations, the protocol was not prospectively registered. This limits external verification of whether all eligibility criteria, extraction procedures, and analytic decisions were specified a priori, and should therefore be considered a methodological limitation.

## 5. Conclusions

This systematic review and meta-analysis suggests that study-defined elevated academic procrastination is frequent among health sciences students. However, the pooled prevalence should not be interpreted as a definitive universal estimate, given the very high between-study heterogeneity observed across the included studies. Differences in geographic region, professional program, sampling methods, measurement instruments, cut-off criteria, and lack of population-specific validation appear to be major contributors to this variability. Therefore, the main contribution of this review is not only the estimation of prevalence but also the identification of important methodological and measurement limitations that affect comparability across studies.

These findings highlight the need to recognize academic procrastination as a relevant academic and psychological phenomenon that may affect students’ performance, mental health, and educational trajectory. Educational institutions should consider strategies for early identification and management, particularly during the initial years of training, including self-regulation programs, time management training, and psychological support. Future research should prioritize longitudinal designs, probabilistic sampling, population-specific validation of instruments, transparent reporting of impairment or distress criteria, and standardized operational definitions and cut-off points to improve the comparability and clinical or educational usefulness of the evidence in this field.

## Figures and Tables

**Figure 1:**
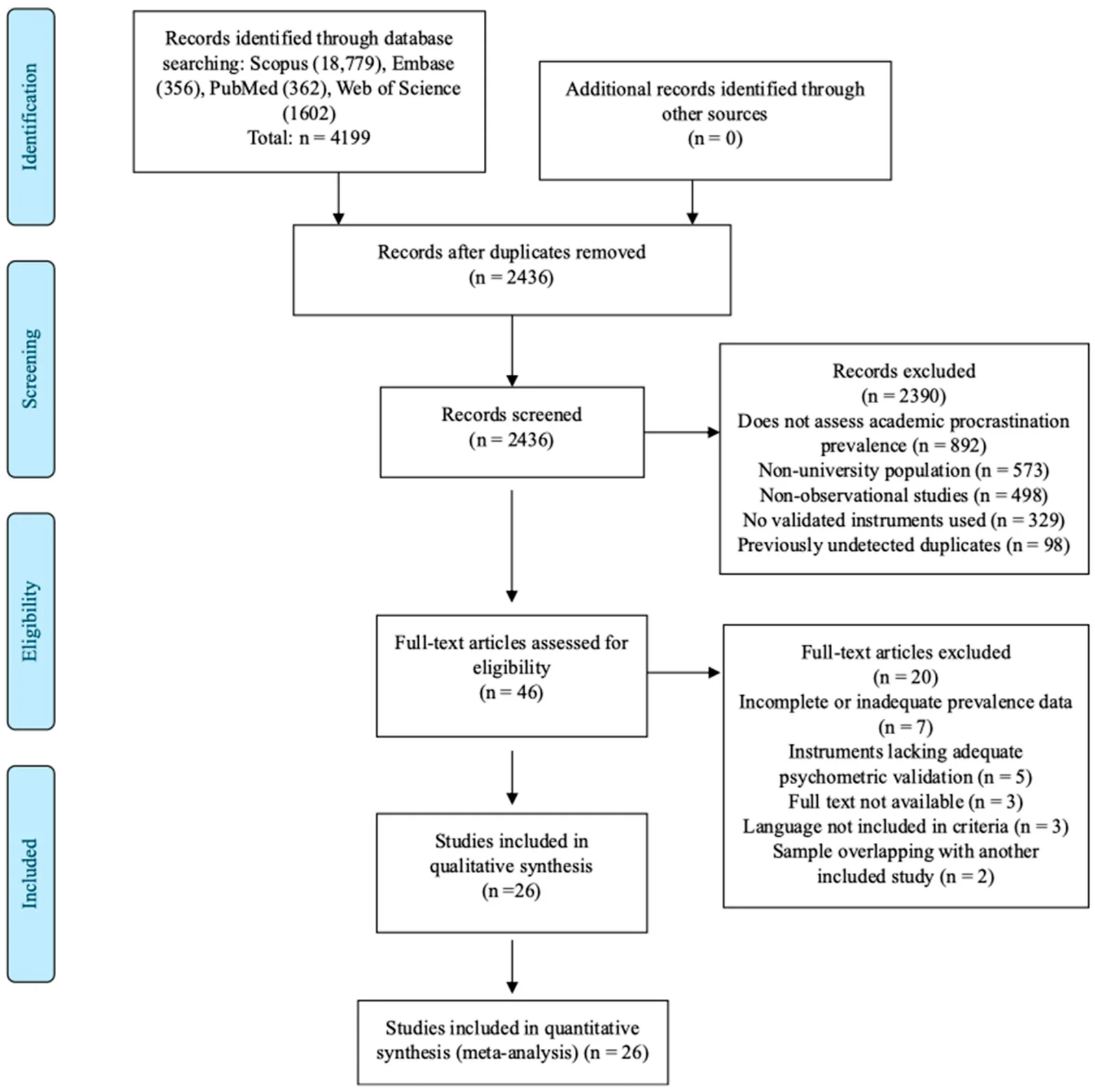
Flowchart of study selection.

**Figure 2:**
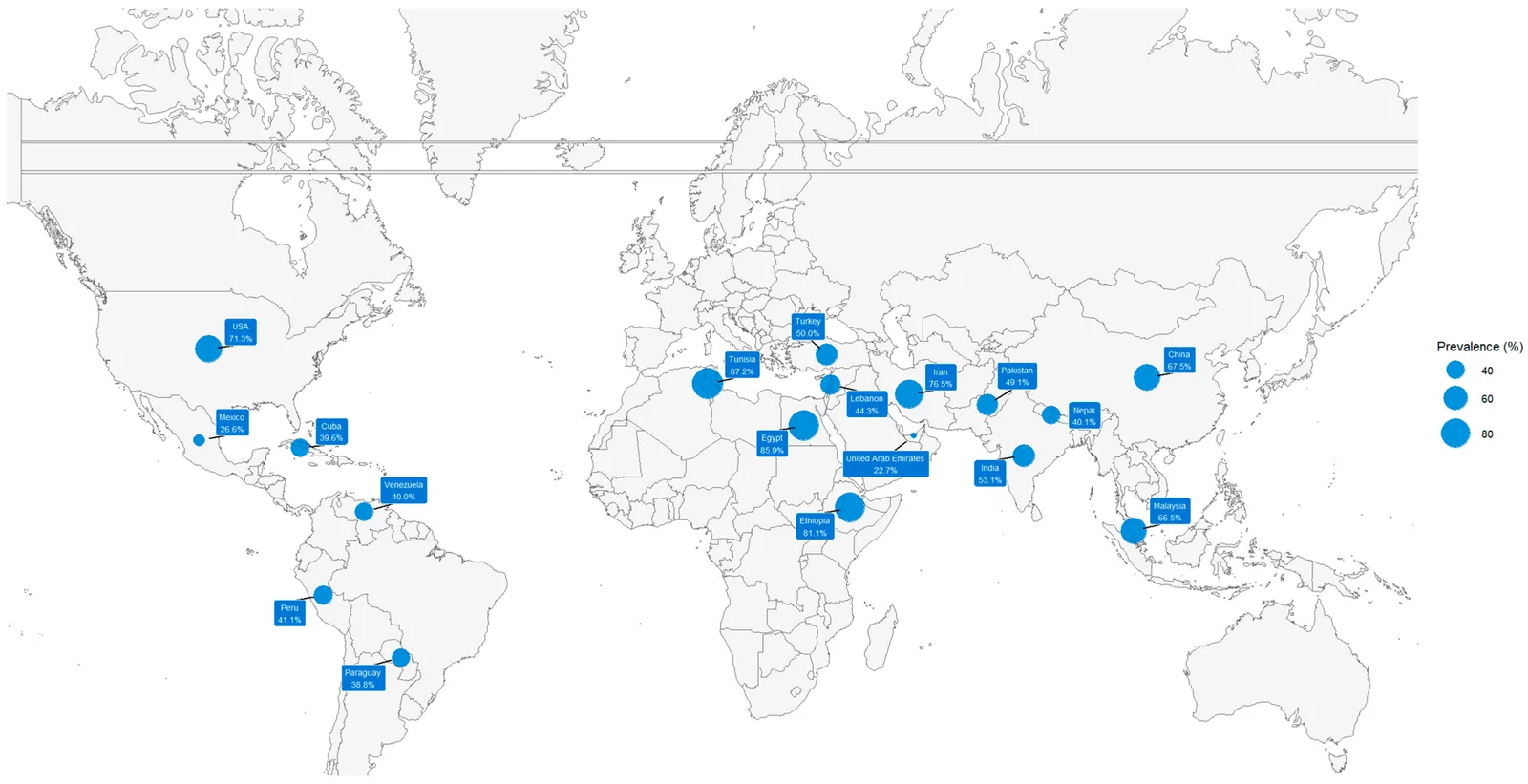
Worldwide distribution of the prevalence of procrastination in health science students. Note: Most studies used instruments validated in previous research or other populations but did not conduct specific psychometric validation for their target study population. Only two studies (Corrales-Reyes et al., 2022; Portillo & Soto, 2024) performed validation specifically for their study populations.

**Figure 3:**
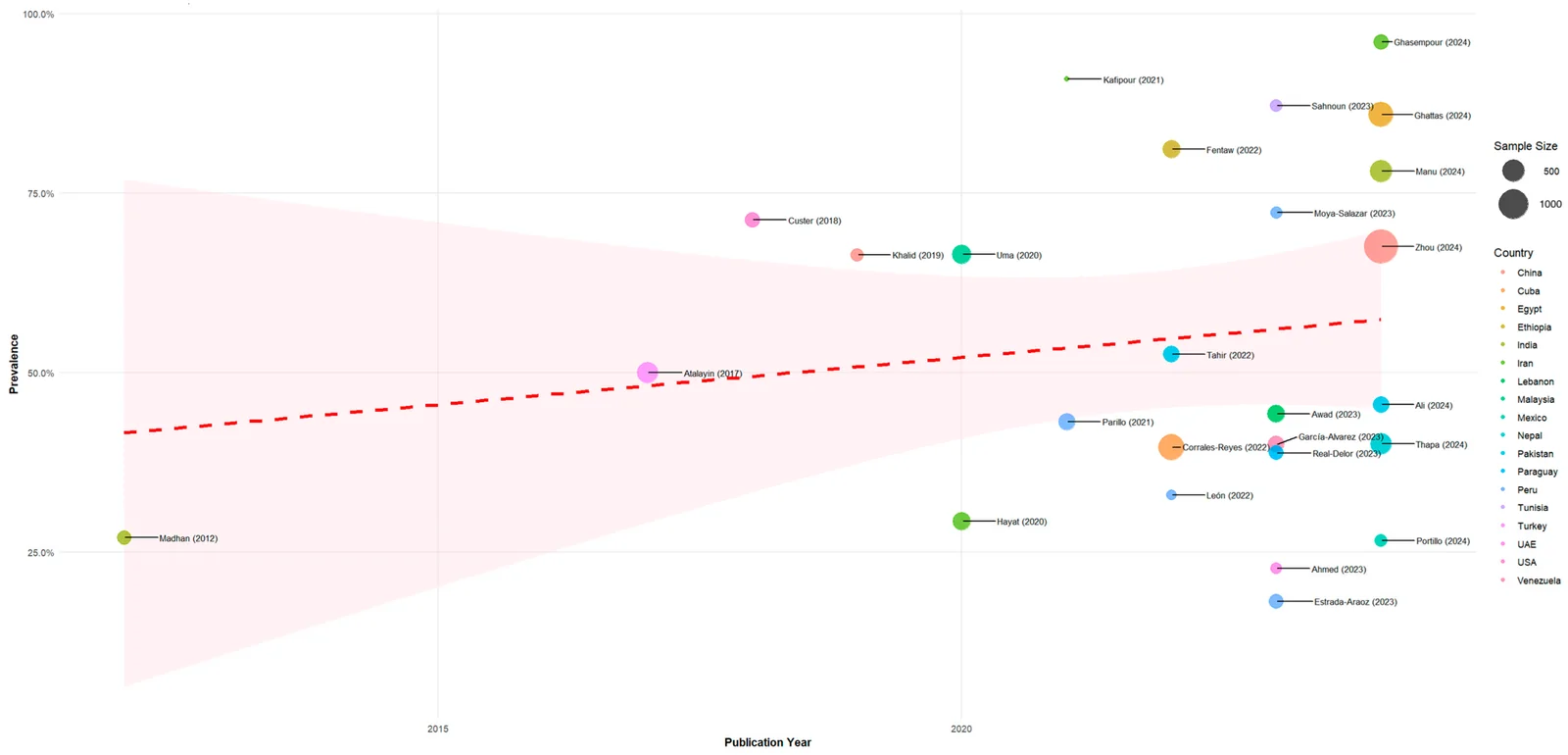
Meta-regression analysis: trend in the prevalence of academic procrastination in health sciences students according to year of publication.

**Table 1:** Meta-analysis of the prevalence of study-defined elevated academic procrastination in health sciences students, according to methodological and population characteristics.

	Number of Studies	Number of Participants	95% CI	I^2^
Prevalence of study-defined elevated academic procrastination	26	8211	55.35 (46.55–63.98)	98%
Sampling design				
Non-probabilistic	24	7776	53.44 (44.30–62.47)	98%
Probabilistic	2	435	77.46 (68.32–85.46)	73%
Measurement instrument				
LPS	2	535	46.58 (11.89–83.31)	99%
TPS-TV	4	1096	65.69 (42.62–85.42)	98%
PASS	10	2325	60.10 (43.34–75.74)	98%
EPA	6	2768	45.38 (29.10–62.17)	98%
APS	3	1363	60.84 (28.43–88.66)	99%
MHCA	1	124	26.61 (19.62–35.01)	―
Type of Career				
Dentistry	4	1725	45.76 (31.64–60.23)	97%
Nursing	8	3074	62.60 (46.88–77.08)	98%
Medicine	12	2834	52.62 (37.63–67.38)	98%
Psychology	2	578	61.67 (20.95–94.42)	99%

**Table 2:** Methodological and psychometric characteristics of the instruments used to assess academic procrastination.

First Author	Year	Country	Instrument Name (Complete and Abbreviated)	Instrument Validated in This Population?	Validation Method Used	Reported Reliability Method	Cut-Off Point Used
Madhan	2012	India	Lay’s Procrastination Scale-student version (LPS)	No	Used Lay (1986) validation but not validated in Indian dental students	Cronbach’s alpha = 0.82	Significant procrastination ≥60 points
Atalayin	2017	Turkey	Tuckman Procrastination Scale—Turkish Version (TPS-TV)	No	Used previous Turkish adaptation but not validated in dental students	Internal consistency and test–retest reliability	Median split (≥40 points)
Custer	2018	USA	Procrastination Assessment Scale for Students (PASS)	No	Used Solomon and Rothblum (1984) validation but not in nursing students	Cronbach’s alpha = 0.84	The study does not report cut-off criteria
Khalid	2019	China	Procrastination Assessment Scale—Students (PASS)	No	Used Solomon and Rothblum (1984) validation but not in Chinese medical students	Not reported	Continuous scale without defining thresholds
Hayat	2020	Iran	Procrastination Assessment Scale for Students (PASS)	No	Used Solomon and Rothblum (1984) validation but not in Iranian medical students	Content validity and face validity by expert panel, Cronbach’s alpha = 0.81	No cut-off points specified
Uma	2020	Malaysia	Lay’s Procrastination Scale for students	No	Used Lay (1986) validation but not in Malaysian dental students	Content validation and face validity by experts, Cronbach’s alpha = 0.765	Original cut-off: high ≥ 62. Main synthesis: moderate + high.
Parillo	2021	Peru	Busko’s Procrastination Scale (EPA)	No	Used Domínguez et al. (2014) Peruvian adaptation but not in nursing students	Cronbach’s alpha = 0.780	The study did not report cut-off criteria
Kafipour	2021	Iran	Procrastination Assessment Scale for Students (abbreviated, 5 items)	No	Used Solomon and Rothblum (1984) validation but not in Iranian medical students	Translation to Persian and validation by TEFL experts, Cronbach’s alpha = 0.75	No specific thresholds provided
Fentaw	2022	Ethiopia	Combination of Tuckman Procrastination Scale (adapted) and own questionnaire	No	Partially adapted Tuckman scale but not validated in Ethiopian health sciences students	Pilot study, Cronbach’s alpha = 0.89	Low < 24 and high > 25 points
Corrales-Reyes	2022	Cuba	Academic Procrastination Scale EPA-11-Odonto	Yes	Specifically validated for Cuban Stomatology students	Cronbach’s alpha = 0.786	Classification based on upper tertile
León	2022	Peru	Academic Procrastination Scale (EPA)	No	Used Domínguez et al. (2014) adaptation but not in Peruvian medical students	Cronbach’s alpha = 0.911	Positive results > 45 and negative 25–44 points
Tahir	2022	Pakistan	Academic Procrastination Scale (APS)	No	Used McCloskey and Scielzo (2015) validation but not in Pakistani medical students	Cronbach’s alpha 0.86 to 0.93 by academic year	Median of total score used as reference
García-Alvarez	2023	Venezuela	Procrastination Assessment Scale for Students (EEPE)	No	Used Spanish adaptation by Natividad (2014) but not in Venezuelan psychology students	Cronbach’s alpha = 0.95	No categories or levels specified
Moya-Salazar	2023	Peru	Academic Procrastination Scale (EPA) (Busko, 1998)	No	Used Domínguez et al. (2014) adaptation but not in Peruvian nursing students during internship	Cronbach’s alpha = 0.766	Scores ≥ 36 interpreted as high levels
Real-Delor	2023	Paraguay	García-Martínez and Silva Questionnaire	No	Used García and Silva (2019) validation but not in Paraguayan medical students	Exploratory factor analysis, Cronbach’s alpha = 0.830	60th percentile (value 2.9) used as threshold
Turki	2023	Tunisia	Tuckman Procrastination Scale (TPS)	No	No specific validation for Tunisia mentioned	Not reported	Score ≥ 80 considered high procrastination
Awad	2023	Lebanon	Procrastination Assessment Scale for Students (PASS)	No	Used Solomon and Rothblum (1984) validation but not in Lebanese medical students	Clarity pilot test, Cronbach’s alpha = 0.880	The study did not define levels or cut-off points
Estrada-Araoz	2023	Peru	Academic Procrastination Scale	No	Used Domínguez et al. (2014) adaptation but not in Peruvian nursing students	Content-based validity (Aiken’s V = 0.922), Cronbach’s alpha = 0.911	Categorized as low (33.2%), moderate (40.1%), and high (26.7%)
Ahmed	2023	United Arab Emirates	Procrastination Assessment Scale for Students (PASS)	No	Used Solomon and Rothblum (1984) validation but not in UAE medical students	Not reported	No classifications based on specific thresholds
Manu	2024	India	Procrastination Assessment Scale (PASS)	No	Used Solomon and Rothblum (1984) validation but not in Indian medical students	Content validity by expert panel, not reported	No numerical references provided
Portillo	2024	Mexico	Academic procrastination scale (MHCA)	Yes	Designed and validated in Mexico with medical students	Cronbach’s alpha = 0.940	Low (1–4), medium (5–6), high (≥7), very high (≥9)
Ghasempour	2024	Iran	Solomon and Rothblum’s Procrastination Assessment Scale—Students (PASS)	No	Used Solomon and Rothblum (1984) validation but not specifically validated in Iranian medical science students	Confirmatory factor analysis (CFA) Cronbach’s alpha = 0.85	Mild (27–62), moderate (63–98), severe (99–135)
Thapa	2024	Nepal	Academic Procrastination Scale (Not abbreviated)	No	Used McCloskey and Scielzo (2015) validation but not in Nepalese students	Cronbach’s alpha = 0.94	Score ≥ 80 indicated high procrastination
Ghattas	2024	Egypt	Academic Procrastination Scale (APS)	No	Used McCloskey and Scielzo (2015) validation but not in Egyptian nursing students	Translation, back-translation, review by 5 experts and Content Validity Ratio (CVR), Cronbach’s alpha = 0.94	Low (25–58), moderate (59–91), high (92–125)
Zhou	2024	China	Academic Procrastination Inventory for College Students	No	Used previous validation in Chinese populations but not specifically in vocational nursing students	Cronbach’s alpha = 0.917	Mild (39–57), moderate (58–76), severe (77–95)
Ali	2024	Pakistan	Procrastination Assessment Scale for Students (PASS)	No	Used Solomon and Rothblum (1984) validation but not in Pakistani nursing students	Content validity and previous studies, Cronbach’s alpha = 0.93	Mild (18–41), moderate (42–65), severe (66–90)

Note: For the meta-analysis, cases were extracted using the operational definition reported in each primary study. In categorical studies, moderate, high, severe, or equivalent categories were coded as cases, while low, mild, absent, or non-procrastination categories were coded as non-cases. This coding reflects study-defined elevated academic procrastination and should not be interpreted as a uniform definition of chronic procrastinator status. In Uma et al. (2020), the original high-only category was 103/361, whereas the numerator used in the main synthesis was 240/361 because it corresponded to moderate + high procrastination under the harmonization rule.

## Data Availability

All data supporting the findings of this study are contained within the article and its Supplementary Materials.

## Supplementary Materials

The following supporting information can be downloaded at https://www.mdpi.com/article/10.3390/ejihpe16100154/s1: Table S1. PRISMA 2020 Checklist. Table S2. Search strategy. Table S3. Main characteristics of the selected studies.

## Abbreviations

PASS (Procrastination Assessment Scale for Students); EPA (Academic Procrastination Scale); TPS-TV (Tuckman Procrastination Scale—Turkish Version); LPS (Lay Procrastination Scale); APS (Academic Procrastination Scale); MHCA (Multifactorial Healthy Cognitive Assessment).
