# Supplementary material for: Measurement Heterogeneity and Pooled Prevalence of Academic Procrastination Among Health Sciences Students: A Systematic Review and Meta-Analysis
Source: Eur J Investig Health Psychol Educ. 2026 Oct 1;16(10):154. doi: 10.3390/ejihpe16100154 (PMC13703183; doi:10.3390/ejihpe16100154)
Supplement: Supplementary file 1 [file ejihpe-16-00154-s001.zip › ejihpe-4340452-supplementary.pdf]

Table S1. PRISMA 2020 checklist.

Section and Topic	Item #	Checklist item	Location where item is reported
**TITLE**			
Title	1	Identify the report as a systematic review.	Page 1
**ABSTRACT**			
Abstract	2	See the PRISMA 2020 for Abstracts checklist.	Page 1
**INTRODUCTION**			
Rationale	3	Describe the rationale for the review in the context of existing knowledge.	Page 2
Objectives	4	Provide an explicit statement of the objective(s) or question(s) the review addresses.	Page 2
**METHODS**			
Eligibility criteria	5	Specify the inclusion and exclusion criteria for the review and how studies were grouped for the syntheses.	Page 3
Information sources	6	Specify all databases, registers, websites, organisations, reference lists and other sources searched or consulted to identify studies. Specify the date when each source was last searched or consulted.	Page 3
Search strategy	7	Present the full search strategies for all databases, registers and websites, including any filters and limits used.	Page 3
Selection process	8	Specify the methods used to decide whether a study met the inclusion criteria of the review, including how many reviewers screened each record and each report retrieved, whether they worked independently, and if applicable, details of automation tools used in the process.	Page 3
Data collection process	9	Specify the methods used to collect data from reports, including how many reviewers collected data from each report, whether they worked independently, any processes for obtaining or confirming data from study investigators, and if applicable, details of automation tools used in the process.	Page 3-4
Data items	10a	List and define all outcomes for which data were sought. Specify whether all results that were compatible with each outcome domain in each study were sought (e.g. for all measures, time points, analyses), and if not, the methods used to decide which results to collect.	Page 4
	10b	List and define all other variables for which data were sought (e.g. participant and intervention characteristics, funding sources). Describe any assumptions made about any missing or unclear information.	Page 4
Study risk of bias assessment	11	Specify the methods used to assess risk of bias in the included studies, including details of the tool(s) used, how many reviewers assessed each study and whether they worked independently, and if applicable, details of automation tools used in the process.	Page 6
Effect measures	12	Specify for each outcome the effect measure(s) (e.g. risk ratio, mean difference) used in the synthesis or presentation of results.	Page 6
Synthesis methods	13a	Describe the processes used to decide which studies were eligible for each synthesis (e.g. tabulating the study intervention characteristics and comparing against the planned groups for each synthesis (item #5)).	Page 3-4
	13b	Describe any methods required to prepare the data for presentation or synthesis, such as handling of missing summary statistics, or data conversions.	Page 4
	13c	Describe any methods used to tabulate or visually display results of individual studies and syntheses.	Page 4
	13d	Describe any methods used to synthesize results and provide a rationale for the choice(s). If meta-analysis was performed,	Page 4

Section and Topic	Item #	Checklist item	Location where item is reported
		describe the model(s), method(s) to identify the presence and extent of statistical heterogeneity, and software package(s) used.	
	13e	Describe any methods used to explore possible causes of heterogeneity among study results (e.g. subgroup analysis, meta-regression).	Page 4
	13f	Describe any sensitivity analyses conducted to assess robustness of the synthesized results.	Page 4
Reporting bias assessment	14	Describe any methods used to assess risk of bias due to missing results in a synthesis (arising from reporting biases).	-----
Certainty assessment	15	Describe any methods used to assess certainty (or confidence) in the body of evidence for an outcome.	-----
**RESULTS**			
Study selection	16a	Describe the results of the search and selection process, from the number of records identified in the search to the number of studies included in the review, ideally using a flow diagram.	Page 5
	16b	Cite studies that might appear to meet the inclusion criteria, but which were excluded, and explain why they were excluded.	Page 5
Study characteristics	17	Cite each included study and present its characteristics.	Page 5-6
Risk of bias in studies	18	Present assessments of risk of bias for each included study.	Page 7-8
Results of individual studies	19	For all outcomes, present, for each study: (a) summary statistics for each group (where appropriate) and (b) an effect estimate and its precision (e.g. confidence/credible interval), ideally using structured tables or plots.	Page 10-11
Results of syntheses	20a	For each synthesis, briefly summarise the characteristics and risk of bias among contributing studies.	Page 5-6
	20b	Present results of all statistical syntheses conducted. If meta-analysis was done, present for each the summary estimate and its precision (e.g. confidence/credible interval) and measures of statistical heterogeneity. If comparing groups, describe the direction of the effect.	Page 7-11
	20c	Present results of all investigations of possible causes of heterogeneity among study results.	Page 9-11
	20d	Present results of all sensitivity analyses conducted to assess the robustness of the synthesized results.	Page 9-11
Reporting biases	21	Present assessments of risk of bias due to missing results (arising from reporting biases) for each synthesis assessed.	Page 9-10
Certainty of evidence	22	Present assessments of certainty (or confidence) in the body of evidence for each outcome assessed.	Page 9-10
**DISCUSSION**			
Discussion	23a	Provide a general interpretation of the results in the context of other evidence.	Page 11-13
	23b	Discuss any limitations of the evidence included in the review.	Page 14
	23c	Discuss any limitations of the review processes used.	Page 14
	23d	Discuss implications of the results for practice, policy, and future research.	Page 13-14
**OTHER INFORMATION**			
Registration and protocol	24a	Provide registration information for the review, including register name and registration number, or state that the review was not registered.	Page 3
	24b	Indicate where the review protocol can be accessed, or state that a protocol was not prepared.	Page 6
	24c	Describe and explain any amendments to information provided at	Page 6

Section and Topic	Item #	Checklist item	Location where item is reported
		registration or in the protocol.	
Support	25	Describe sources of financial or non-financial support for the review, and the role of the funders or sponsors in the review.	Page 1
Competing interests	26	Declare any competing interests of review authors.	Page 17
Availability of data, code and other materials	27	Report which of the following are publicly available and where they can be found: template data collection forms; data extracted from included studies; data used for all analyses; analytic code; any other materials used in the review.	Page 17

Adapted from Page, M. J., McKenzie, J. E., Bossuyt, P. M., Boutron, I., Hoffmann, T. C., Mulrow, C. D., et al. (2021). The PRISMA 2020 statement: An updated guideline for reporting systematic reviews. BMJ, 372, n71. https://doi.org/10.1136/bmj.n71. Licensed under CC BY 4.0.

Table S2. Search strategy.

Search strategy in PUBMED	
#1	"procrastination"[mesh] OR "procrastination"[tiab] OR "academic procrastination"[tiab]
#2	"Students"[Mesh] OR "Students, Medical"[Mesh] OR "medical student"[tiab] OR "medical students"[tiab] OR "college student"[tiab] OR "college students"[tiab] OR "university student"[tiab] OR "university students"[tiab] OR "undergraduate student"[tiab] OR "undergraduate students"[tiab]
#3	#1 AND #2
Search strategy in SCOPUS	
#1	TITLE-ABS-KEY ("procrastination" OR "academic procrastination")
#2	TITLE-ABS-KEY ("medical student" OR "medical students" OR "college student" OR "college students" OR "university student" OR "university students" OR "undergraduate student" OR "undergraduate students" OR "students, medical" OR "students")
#6	#1 AND #2
Search strategy in Web of Science	
#1	TS=("procrastination" OR "academic procrastination")
#2	TS=("medical student" OR "medical students" OR "college student" OR "college students" OR "university student" OR "university students" OR "undergraduate student" OR "undergraduate students" OR "students, medical" OR "students")
#3	#1 AND #2
Search strategy in EMBASE	
#1	procrastination:ti,ab OR 'academic procrastination':ti,ab OR 'procrastination'/exp
#2	'student'/exp OR 'medical student'/exp OR 'medical student':ti,ab OR 'medical students':ti,ab OR 'college student':ti,ab OR 'college students':ti,ab OR 'university student':ti,ab OR 'university students':ti,ab OR 'undergraduate student':ti,ab OR 'undergraduate students':ti,ab
#3	#1 AND #2

Table S3. Main characteristics of the selected studies.

First Author	Year	Country	Sampling	Selection Criteria Summary	Sample	Sex (% female)	Age	n	Risk of Bias	Instrument (acronym)
Madhan	2012	India	Non-probabilistic	Fourth-year dentistry students from three universities in India with voluntary participation	174	68.96 (120/174)	22.85	47	8 points (Low)	LPS
Atalayin	2017	Turkey	Non-probabilistic	Dentistry students from Ege University in Turkey who voluntarily agreed to participate in the study	452	60.4 (273/452)	20.75	226	8 points (Low)	TPS-TV
Custer	2018	USA	Non-probabilistic	Students from nursing programs (diploma, associate, and bachelor's) pre-licensure in Pennsylvania	202	83.20%	23.9	144	8 points (Low)	PASS
Khalid	2019	China	Non-probabilistic	First-year medical students	140	55.70%	Not specified	93	8 points (Low)	PASS
Hayat	2020	Iran	Non-probabilistic	Medical students from Shiraz University of Medical Sciences with voluntary participation. Excluded: those with incomplete responses or without consent	317	42.6 (135/317)	Not specified	93	8 points (Low)	PASS
Uma	2020	Malaysia	Non-probabilistic	Undergraduate students in dentistry	361	70.40%	Not specified	240	8 points (Low)	LPS
Parillo	2021	Peru	Non-probabilistic	Nursing students from first to fifth year	271	92.98%	Not specified	117	8 points (Low)	EPA
Kafipour	2021	Iran	Non-probabilistic	Medical students with mandatory English writing course, semester 2 (2016)	44	68.20%	Not specified	40	8 points (Low)	PASS

Fentaw	2022	Ethiopia	Probabilistic	Health sciences students from universities in the Amhara region, Ethiopia. Excluded: incomplete responses.	323	51.9 (168/323)	21.6	262	8 points (Low)	TPS-TV
Corrales-Reyes	2022	Cuba	Non-probabilistic	Stomatology students from 7 Cuban universities	738	63.69%	21.67	292	8 points (Low)	EPA
León	2022	Peru	Non-probabilistic	Second-year medical students	91	59.34%	21.68	30	8 points (Low)	EPA
Tahir	2022	Pakistan	Non-probabilistic	Preclinical medical students (1st to 3rd year); clinical excluded	255	Not reported	Not specified	134	8 points (Low)	APS
García-Alvarez	2023	Venezuela	Non-probabilistic	Psychology students, of legal age, with active enrollment in the university	255	71.37 (182/255)	21.24	102	8 points (Low)	PASS
Moya-Salazar	2023	Peru	Probabilistic	Nursing students in internship (fifth year). Excluded: incomplete responses, chronic diseases, or pregnancy	112	88.4 (99/112)	29	81	8 points (Low)	EPA
Real-Delor	2023	Paraguay	Non-probabilistic	Medical students from public and private universities in Paraguay (2022)	188	64.9	24	73	8 points (Low)	TPS-TV
Turki	2023	Tunisia	Non-probabilistic	Medical students in Tunisia. Hedi Chaker University of Sfax	133	81.80%	26	116	8 points (Low)	TPS-TV
Awad	2023	Lebanon	Non-probabilistic	Medical students, ≥18 years, full-time	296	56.10%	22.41	131	8 points (Low)	PASS
Estrada-Araoz	2023	Peru	Non-probabilistic	Nursing students	187	68.40%	Not specified	34	8 points (Low)	EPA

Ahmed	2023	United Arab Emirates	Non-probabilistic	Medical students who consented and completed the questionnaire	110	67.50%	Not specified	25	8 points (Low)	PASS
Manu	2024	India	Non-probabilistic	Medical students included in a cross-sectional study; specific eligibility criteria were not clearly reported	521	56 (294/521)	Not specified	407	7 points (Moderate)	PASS
Portillo	2024	Mexico	Non-probabilistic	New medical students at the Faculty of Higher Studies Iztacala, UNAM. Excluded: incomplete, biased, or repetitive responses	124	70 (87/124)	18.9	33	8 points (Low)	MHCA
Ghasempour	2024	Iran	Non-probabilistic	Medical science students, without serious psychiatric history or recent stressful events	205	64.40%	22.4	197	8 points (Low)	PASS
Thapa	2024	Nepal	Non-probabilistic	Mixed health sciences	454	51.80%	22.1	182	8 points (Low)	APS
Ghattas	2024	Egypt	Non-probabilistic	Nursing students in emergency course (University of Alexandria)	654	83.50%	Not specified	562	8 points (Low)	APS
Zhou	2024	China	Non-probabilistic	Nursing students	1369	86.00%	19.8	925	8 points (Low)	EPA
Ali	2024	Pakistan	Non-probabilistic	GBSN nursing students (1st to 4th year), volunteers	235	10.60%	21.41	107	8 points (Low)	PASS

Legend: PASS: Procrastination Assessment Scale for Students; EPA: Academic Procrastination Scale; TPS-TV: Tuckman Procrastination Scale—Turkish Version; LPS: Lay Procrastination Scale; APS: Academic Procrastination Scale; MHCA: Multifactorial Healthy Cognitive Assessment. The "n" column indicates the number of students with academic procrastination in each study according to the harmonization rule used in the main meta-analysis. For Uma et al. (2020), n = 240 corresponds to the moderate + high procrastination categories coded as study-defined elevated academic procrastination; the original high-only category

was 103/361. Risk of bias was evaluated using a point-based scale and classified as follows: low risk = 8–10 points, moderate risk = 5–7 points, and high risk = ≤4 points.
